# Assessing crowd management strategies for the 2010 Love Parade disaster using computer simulations and virtual reality

**DOI:** 10.1098/rsif.2020.0116

**Published:** 2020-06-10

**Authors:** Hantao Zhao, Tyler Thrash, Mubbasir Kapadia, Katja Wolff, Christoph Hölscher, Dirk Helbing, Victor R. Schinazi

**Affiliations:** 1 Chair of Cognitive Science, ETH Zürich, Zurich, Switzerland; 2 Interactive Geometry Lab, ETH Zürich, Zurich, Switzerland; 3 Computational Social Science, ETH Zürich, Zurich, Switzerland; 4 Geographic Information Visualization and Analysis, University of Zürich, Zurich, Switzerland; 5 Digital Society Initiative, University of Zürich, Zurich, Switzerland; 6 Department of Computer Science, Rutgers University, Piscataway, NJ, USA; 7 Department of Psychology, Bond University, Gold Coast, Queensland, Australia

**Keywords:** crowd simulation, crowd disasters, virtual reality, spatial cognition, physiological arousal

## Abstract

Dense crowds in public spaces have often caused serious security issues at large events. In this paper, we study the 2010 Love Parade disaster, for which a large amount of data (e.g. research papers, professional reports and video footage) exist. We reproduce the Love Parade disaster in a three-dimensional computer simulation calibrated with data from the actual event and using the social force model for pedestrian behaviour. Moreover, we simulate several crowd management strategies and investigate their ability to prevent the disaster. We evaluate these strategies in virtual reality (VR) by measuring the response and arousal of participants while experiencing the simulated event from a festival attendee’s perspective. Overall, we find that opening an additional exit and removing the police cordons could have significantly reduced the number of casualties. We also find that this strategy affects the physiological responses of the participants in VR.

## 1. Introduction

Crowd disasters during large-scale events are a primary concern for event security because of the related casualties and chaos. However, the investigation of conditions that lead to crowd disasters in real environments is often infeasible because of practical and ethical issues. By contrast, computer simulations can contribute to our understanding of crowd disasters by providing a framework for the formal analysis of an event. In addition, virtual reality (VR) experiments allow researchers to investigate individuals’ responses to simulated crowds and the physical and social conditions surrounding the event (e.g. exiting barriers or fences) and to precisely measure participants’ physiological reactions and spatial behaviour. Both simulation and VR approaches may facilitate the development of disaster prevention methods.

The goal of this paper is to employ a combination of simulation and VR methods to gain a better understanding of crowd management and to help organizers avoid similar disasters in the future. Specifically, we investigate interventions that might have been able to prevent the disaster at the 2010 Love Parade music festival in Duisburg, Germany. First, we reproduce the events of the 2010 Love Parade disaster with a simulation based on the social force model (SFM) [1] calibrated with available data (https://loveparade2010doku.wordpress.com/). We then test several possible crowd management strategies, including the removal of physical obstacles and the separation of inflow and outflow. These strategies are evaluated with respect to crowd density, throughput, congestion and the number of simulated casualties. While the results of this simulation appear to match observations from the actual event, our approach focuses on the density of the event within a simplified crowd behaviour framework. More sophisticated models incorporating local behaviours are possible and can be the focus of future research. Second, we conduct a VR experiment in order to investigate differences in the simulated first-person experiences of the original disaster and the best-performing crowd management strategy using a head-mounted display. Here, individual participants are immersed in one of two crowd scenarios as we measure their physiological arousal and self-reported level of stress.

### 1.1. Computer simulations of crowd behaviour

Many researchers have attempted to address the conditions that lead to crowd disasters using computer simulations [2–5], real-world observations [6–8], real-world experiments [9] and VR experiments [10]. Computer simulations of real events have been used to study crowd disasters because of their versatility and relatively low cost. Simulations provide the ability to predict crowd behaviour in new and unseen environmental conditions, allowing researchers to conduct ‘what if’ experiments [4]. Computer simulations typically employ steering algorithms for individual agents and are evaluated with respect to the behaviour of a large number of agents [5]. For example, the SFM describes the self-organization of pedestrian movement using a microscopic model of pedestrians [1,3]. Inspired by the Newtonian law of motion, this model has been successful in reproducing several common crowd phenomena, such as lane formation and crowd turbulence [11]. In order to evaluate the outcome of the simulations, density [2], congestion [12] and crowding [13] have been used as metrics to assess the level of risk.

### 1.2. Real-world observations and experiments

Based on observations of the Love Parade disaster, a number of studies have begun to examine the management of the event [7,14]. In order to prevent future disasters, Helbing & Mukerji [7] have suggested the separation of inflow and outflow, the removal of obstacles (e.g. fences, police cordons) and the provision of additional entrances/exits. Klüpfel [14] has assessed the underlying causes and consequences of the Love Parade disaster and has suggested that the proximate causes of overcrowding include the late opening of the entrance. Lian *et al.* [15] focus on extracting pedestrian movement patterns from publicly available video footage of the disaster. Krausz & Bauckhage [16] extend this work by using the video footage to automatically detect the timing of the congestion. Pretorius *et al.* [4] simulate several management strategies in a model of the Love Parade disaster and find that a one-directional flow might have prevented injury compared with the original event.

In general, data corresponding to real-world events are often difficult to obtain and may violate individuals’ privacy. In comparison, experiments in real environments can be costly and difficult to organize, especially for large crowds, and their scope is limited to situations that do not endanger health or lives. Nonetheless, researchers have developed crowd behaviour detection and flow computation [8] methods that are capable of capturing aggregate behaviour without tracking specific individuals. By applying a similar approach, Moussaïd *et al.* [17] have analysed the organization of social groups to predict walking patterns from video footage. Laboratory experiments have also been used to study local crowd movement patterns at critical regions such as turning corners [18,19] or stairs [20]. For example, Dias *et al.* [19] observed crowd turning behaviour and found that higher turning angles can reduce flow rates and velocities under normal congestion. Similarly, Burghardt *et al.* [20] found that areas of high density can precede a turning point at stairs, where congestion forms. Such empirical evidence can be further used to calibrate data-driven models for pedestrian simulations. Dias & Lovreglio [21] represented the floor as a continuous field in order to better model pedestrians’ navigation of a corner and validated these field representations with the observation of walking behaviour during a laboratory experiment. In addition, Crociani *et al.* [22] proposed an algorithm to reproduce smooth trajectories at corners and validated this algorithm with data from laboratory experiments.

Real-world data from crowd disasters (e.g. during a Hajj event in Mina [2]) can be used to calibrate and validate computer simulations in order to help predict future disasters. Extracting continuous crowd movements from segmented video clips has been a major challenge for acquisition of crowd data from cameras. Khan *et al.* [23] successfully used an unsupervised clustering algorithm to generate crowd flows from segmented video clips and then compared these with other tracking techniques from the literature. Automatic tracking techniques for coarse-grained data analysis can also benefit from reflective markers carried by crowd members [9], and some researchers have employed a more traditional approach by manually extracting data from videos in order to improve head-counting methods [24].

### 1.3. Virtual reality experiments

Compared with real-world experiments, VR studies of crowd behaviour allow for greater experimental control [25] and opportunities for crowd visualization [26]. This type of visualization provides a first-person perspective that can guide the organizers towards better decisions and help patrons to experience the scenario within the crowd. When conducting single-user studies, VR researchers must consider the manner in which the crowd is visualized [26]. Depending on the application, these visualized crowds may need to be representative (e.g. with human-like bodies and movements [27]) or realistic (e.g. with human-like faces and clothing [28]). Despite the opportunities provided by VR for experimental control, crowd visualization and multimodal assessment, there are a few notable limitations. These limitations include, but are not limited to, constraints on task complexity (e.g. the number of turns along a route) [10], motion sickness [29], lack of interaction with real social agents [30] and the lack of real-time proprioceptive feedback (e.g. collisions between avatars) [25].

### 1.4. Crowds and physiological arousal

Crowds may influence individual self-reported affective [31], behavioural [32] and physiological states [33]. In terms of behavioural effects, virtual crowds can be used positively as a social signal for finding an unobstructed exit [27] or negatively as an obstacle blocking a potential path to safety. Realistic crowd visualization in VR also provides opportunities to investigate changes in psychological states resulting from dangerous scenarios such as crowd disasters. For example, the presence of a standing avatar has been found to lead to a higher physiological arousal than the presence of a running avatar [34], and the appearance [35] and distance [36] of virtual avatars have been found to positively correlate with physiological arousal. Physiological responses such as electrodermal activity (EDA) [37] and heart rate variability (HRV) [38] have been used to study stress/distress [39], user experience [40], attention and other aspects of cognition [41]. EDA and HRV both reflect the activity of the autonomic nervous system. While EDA reflects sympathetic arousal, (normal) HRV results from a balance between sympathetic and parasympathetic activity [38]. Questionnaires can also be used to distinguish between affective states [42].

### 1.5. Overview of the Love Parade crowd disaster

**Figure 1.**
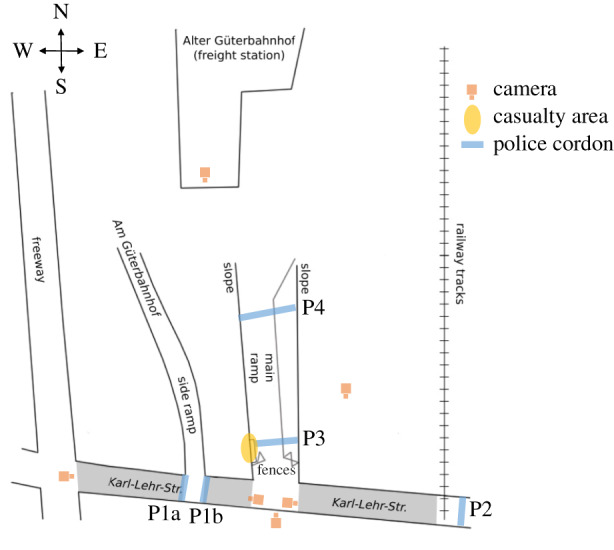
Illustration of the festival area, including the locations of the surveillance cameras used for the simulations, the police cordons (P1–P4) and the casualty area. Adapted from an image created by Helbing & Mukerji [7].

The Love Parade was a popular German music festival that was first organized in 1989. The Love Parade disaster occurred in Duisburg on 24 July 2010 [7]. The festival area was approximately 100 000 m^2^ and was constrained by railway tracks to the east and by a freeway (major road) to the west (figure 1). A tunnel from an old freight station funnelled visitors to a main ramp that led to the festival area. The narrowest diameter of the tunnel was 20 m. The festival area could only be entered and exited through this tunnel, although a side ramp was available as an additional (reserve) exit. The main ramp was 26 m wide at its narrowest point, but with a local effective width of only 10.59 m owing to the presence of temporary fences. The entire festival area was surrounded by fences.

A detailed analysis of the festival area revealed potential safety issues in the event planning, including the limited maximum capacity and the use of the tunnel as the only entrance and exit [7]. The use of the main ramp was temporarily restricted by police cordons that were added to control traffic at 15.50 during the festival. Around this time (between 15.30 and 16.00), congestion around the main ramp began to form. As congestion increased, people began climbing fences, billboards and poles in order to escape from the dense crowd. The fences were removed at approximately 16.20 in an attempt to relieve the congestion [7]. This, however, could not prevent the crowd disaster, which resulted in the death of 21 people and injury to 500 festival attendees. The primary cause of death was suffocation.

## 2. Simulation and interventions

### 2.1. Love Parade crowd disaster simulation

**Figure 2.**
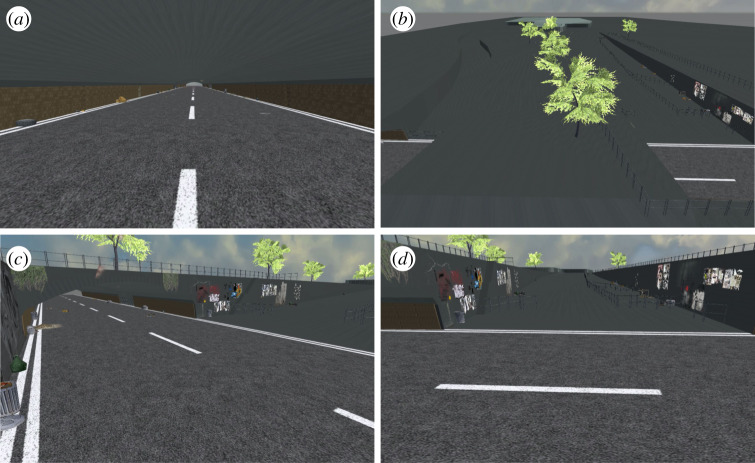
Four different views of the virtual environment. (*a*) View of the tunnel. (*b*) View of the overall area with both the main and side ramps. (*c*) View of the tunnel and a narrow staircase from the entrance to the main ramp. (*d*) View of the main ramp from a similar position to surveillance camera 13.

To simulate the 2010 Love Parade disaster, we use publicly available online data from video surveillance cameras (https://loveparade2010doku.wordpress.com/), a three-dimensional model of the festival area and computer-controlled agents to represent the moving crowd. We then extend this simulation by modelling various crowd management scenarios and compare these simulations in terms of congestion and simulated casualties. We use the Unity 3D Game Engine (http://www.unity.com) to construct a true-to-scale three-dimensional virtual environment of the festival area and all potential entrances and exits (figure 2). This environment was created based on the description from Helbing & Mukerji [7] and publicly available maps, plans and other documents. For the simulation, we use a simple version of the environment without lighting and texture details. The texture and lighting were later added for the VR experiment and modelled based on the surveillance videos (i.e. https://loveparade2010doku.wordpress.com/) of the real environment.

We compute a triangulated representation of the walkable areas in the environment to form a navigation mesh. The starting point and the destinations of the crowd flows were determined from the surveillance videos. A search graph was constructed on the navigation mesh (nodes are the centroids of triangles, and edges connect adjacent triangles). The A* search algorithm [43] was used to perform path-finding operations on the search graph to find sequences of way points between origins and destinations of agents. The SFM [3] was used to steer the agent along this computed path while avoiding collisions with the environment and other pedestrians. Details of the exact targeting mechanism can be found in the electronic supplementary material.

The analysis of the surveillance videos also provided estimates of inflow, outflow, density and individual velocities at different times. Videos from surveillance cameras were used instead of videos from visitors’ mobile phones because they were more stable over time and often of higher quality. Given the availability of the data, each surveillance video was separated into segments of 20 minutes except for the first three segments (7, 6 and 17 min, respectively). Because of technical problems with the recording, some frames were missing, and the effective length of the video segments were shorter than the duration they represented by up to 4 minutes.

In order to estimate visitors’ velocities in metres per second (V(t)) as a function of density (*ρ*(*t*)), we used the fundamental diagram proposed by Weidmann [44],

2.1 $$V(t)=1.34\times (1-{\text{e}}^{-1.913\cdot (1/\rho (t))-1/5.4}).$$

Density can also be estimated by the combined inflow and outflow per second and metre (Q(*t*)) divided by velocity (V(t)),

2.2 $$\rho (t)=\frac{Q(t)}{V(t)}.$$

The solutions of equation (2.1) can be determined numerically using Newton’s method [45]. We used the velocity of the crowd after 15.20, which remains at 0.7 m s^−1^ constantly during the simulation. We computed the flow values for the centre of each time interval based on the total width of the main ramp (irrespective of the fences).

Based on these estimates, we generated a crowd of agents to populate the virtual environment and to simulate the effects of different crowd management scenarios in terms of density, congestion, throughput and casualties. Similar to the real event, the agents entered the main ramp from inside the tunnel and exited from the end of the main ramp towards the festival area. The simulations represented estimates of the real event from 15.20 to 16.40.

The SFM represents systematic forces (i.e. attraction and repulsion) exerted by targets, obstacles and other pedestrians that influence the agents’ movements [3]. Specifically, for agent *α*, these forces include an acceleration force $f_{\alpha }^{0}(v_{\alpha })$, a repulsive force ***f***_*αi*_(***r*_*α*_**) caused by obstacles and boundaries, and repulsive interactions ***f***_*αβ*_(***r*_*α*_**, ***v*_*α*_**, ***r*_*β*_**, ***v*_*β*_**) between the agents [3]. The index of obstacles is *i*, and *β* represents the other agents

2.3 $$f=f_{\alpha }^{0}(v_{\alpha })+f_{\alpha B}(r_{\alpha })+\sum_{\beta (\neq \alpha )}f_{\alpha \beta }(r_{\alpha },v_{\alpha },r_{\beta },v_{\beta })+\sum_{i}f_{\alpha i}(r_{\alpha },r_{i},t).$$

The acceleration force $f_{\alpha }^{0}(v_{\alpha })$ is defined by the direction of the next destination ***e*_*α*_** , desired speed $v_{\alpha }^{0}$ and the current speed ***v*_*α*_** , according to

2.4 $$f_{\alpha }^{0}(v_{\alpha })=\frac{1}{\tau_{\alpha }}(v_{\alpha }^{0}e_{\alpha }-v_{\alpha }).$$

Other obstacles *i* (i.e. fences and walls) define the repulsive forces. In equation (2.5), $r_{\alpha }-r_{i}^{\alpha }$ is the distance between an agent and the obstacle, and *V*_*i*_ represents a potential repulsive force

2.5 $$f_{\alpha i}(r_{\alpha })=-\nabla_{r_{\alpha }}V_{i}(∥r_{\alpha }-r_{i}^{\alpha }∥).$$

Repulsive forces between the agents are defined as follows:

2.6 $$f_{\alpha \beta }=A_{\alpha }\exp\left[\frac{\left(r_{\alpha \beta }-d_{\alpha \beta }\right)}{B_{\alpha }}\right]n_{\alpha \beta }.$$

$A_{\alpha }$ represents interaction strength and $B_{\alpha }$ is the range of the repulsive interactions. *d*_*αβ*_ is the distance between the centres of the mass of agent *α* and *β* and *r*_*αβ*_ is the sum of their radii *r*_*α*_ and *r*_*β*_. ***n***_*αβ*_ represents the normalized vector pointing from agent *β* to *α*. We used the parameters *A*_*α*_ = 0.045, *B*_*α*_ = 0.2, *r*_*α*_=*r*_*β*_=1 and $v_{\alpha }^{0}=1.3\;{\text{m s}}^{-1}$.

### 2.2. Simulation of intervention scenarios

**Figure 3.**
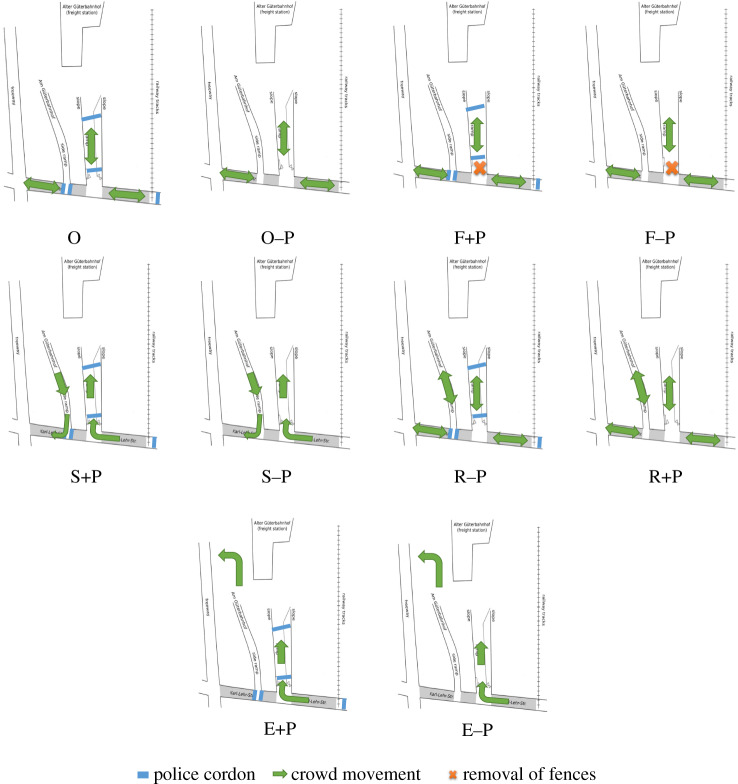
Illustration of the 10 simulated scenarios. Green arrows represent the in- and outflow of the crowd. Blue lines represent the police cordons, and the orange crosses represent the removal of the main fences along the main ramp. Scenario O represents the original simulation; the F scenarios represent the removal of fences; the S scenarios represent the separation of inflow and outflow; the R scenarios represent the inclusion of the side ramp; and the E scenarios represent opening the additional exit. Each of these intervention scenarios has two conditions, either with (+) or without (−) police cordons (P).

After the simulation of the original crowd disaster, we simulated nine alternative crowd management scenarios based on the recommendations of Helbing & Mukerji [7]. These nine scenarios were based on five different variations of the original simulation. First, we varied the presence of the police cordons. The original police cordons were simulated using obstacles that could not be walked through. The idea was that removing the police cordons might reduce crowd density by removing unnecessary obstacles. Second, we varied whether the main fences were present at the beginning of the simulation because the fences may have been unnecessary obstacles increasing crowd density. Third, we varied whether the side ramp was open. We expected that this might reduce the density by decreasing the number of people attempting to move through the same channel. Fourth, we varied whether inflow and outflow were separated or not using both ramps or just the main ramp. The separation of inflow and outflow can avoid confrontations of opposite flow directions. Fifth, we varied whether there was an additional exit near the festival area because this might remove the limitations related to the restricted width of the ramps. In total, we devised 10 scenarios (figure 3): the original simulation (O), the original simulation without police cordons (O–P), the removal of fences while police cordons were present (F+P), the removal of fences and of police cordons (F–P), the separation of inflow and outflow in the presence of police cordons (S+P), the separation of inflow and outflow without police cordons (S–P), the inclusion of the side ramp while police cordons were present (R+P), the opening of the side ramp and no police cordons (R–P), use of an additional exit with police cordons (E+P) and an additional exit and no police cordons (E–P).

These scenarios were each repeated 10 times (conducted simultaneously on various computing units with the same simulation program) and compared in terms of maximum occupation, simulated casualties, throughput, general crowd density near the main ramp and congestion. Consistent with the literature [7], we assumed the possibility of casualties when crowd density exceeded four agents per square metre (i.e. danger zones; D_4_). Since conditions with four agents per square metre are not necessarily deadly, we additionally determined extreme danger zones (D_6_), where the crowd density exceeded six agents per square metre, although other researchers have defined danger zones differently [46]. The throughput was considered to be the number of agents that successfully exited the festival area over the course of the entire simulation. General crowd density was computed as the number of agents per square metre, recorded once every simulated minute and averaged over 80 minutes. Congestion was defined as the number of agents who did not move at least 1 m in 60 s. Cronbach’s *α* was computed for each of these dependent measures in order to assess its consistency across repetitions. We also conducted 2 (with or without police cordons) by 5 (scenario) between-group ANOVAs in order to test for systematic differences among the scenarios in terms of each dependent measure.

### 2.3. Simulation results

**Figure 4.**
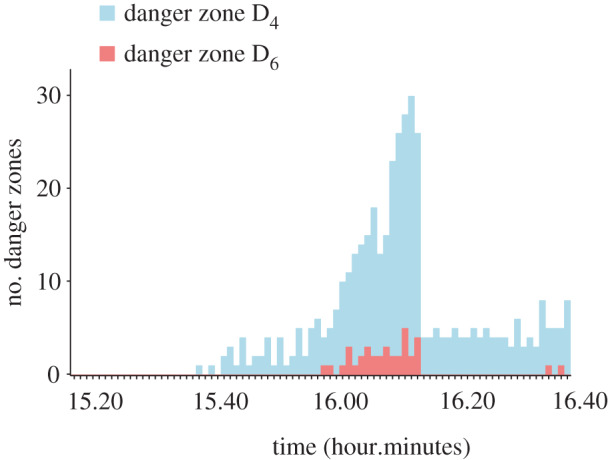
Change in the number of danger zones (D_4_ and D_6_) for the first repetition of the O simulation from 15.20 to 16.40. The number of danger zones increases over time until 16.15 and then suddenly decreases because of the removal of the main fence. This graph peaked at 27 danger zones around 16.15.

**Figure 5.**
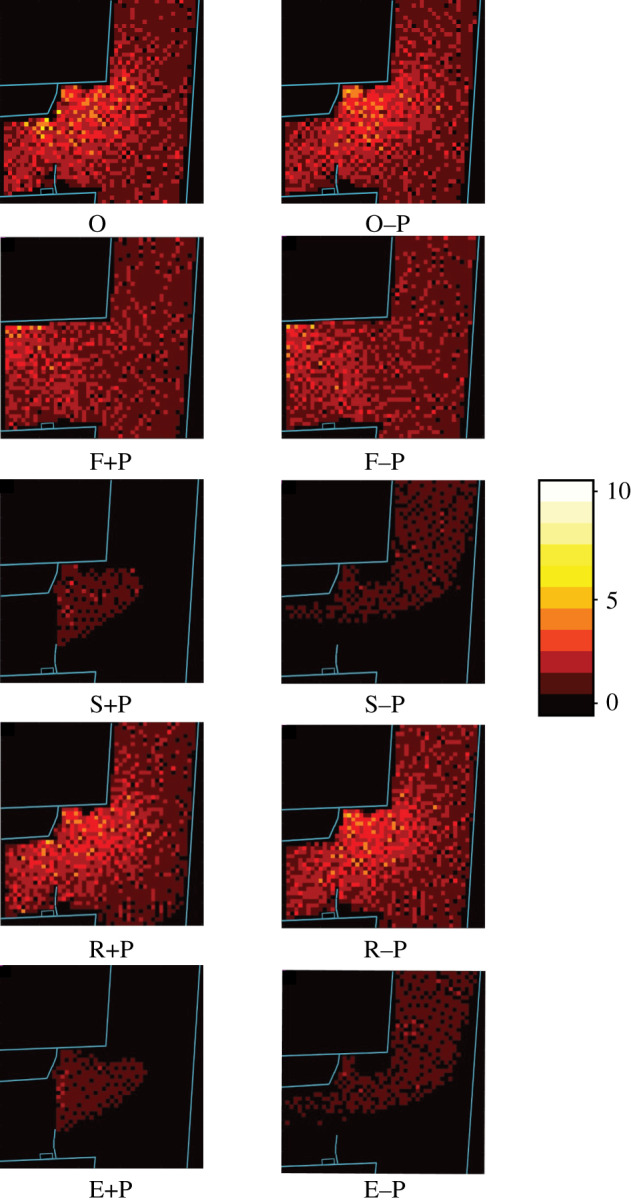
Density maps of the general ramp area at 16.00 for the first repetition of each scenario. The blue lines represent walls and fences. The red dots represent the numbers of pedestrians in each grid cell. The colour bar on the right reflects the number of people in each cell.

Overall, there were very few unrealistically high densities in these simulations. Numerically, all of the alternative crowd management scenarios led to less density, less congestion, more throughput and fewer expected casualties than the simulations of the original event (table 1). There was also extremely high consistency across repetitions for each dependent measure (Cronbach’s *α* > 0.989). The removal of police cordons resulted in the largest differences compared with the original scenario in terms of the reduction in congestion and danger zones. The removal of fences and the use of the side ramp also reduced the number of danger zones, but the most effective scenarios were those that separated inflow and outflow or added another exit near the festival area. These observations were confirmed by a series of ANOVAs. The ANOVA for all dependent measures revealed a significant main effect of police cordons, a significant main effect of scenario and a significant interaction (table 2). The assumption of homogeneity was violated for each ANOVA, so we confirmed each result using a non-parametric aligned ranks transformation ANOVA [47]. In addition, figure 4 represents the first repetition of the O scenario in terms of the number of danger zones over time, and figure 5 illustrates the crowd densities for all 10 scenarios around the accident area at 16.00.

**Table 1.** Means and standard deviations (in parentheses) of simulation results for all 10 replications. Scenario O represents the original simulation; the F scenarios represent the removal of fences; the S scenarios represent the separation of inflow and outflow; the R scenarios represent the inclusion of the side ramp; and the E scenarios represent opening the additional exit. Each of the intervention scenarios has two conditions, with (+) or without (−) police cordons (P). The measures reported here include maximum occupation (max), simulated casualties (D_4_ and D_6_), throughput (TP), general crowd density (density) near the main ramp and congestion. Cronbach’s *α* represents the consistency of these measures across the 10 replications.

scenario	max	D_4_	D_6_	TP	density	congestion
**O**	11.1 (1.8)	357.8 (39.2)	28.7 (6.8)	13.1 (9.7)	0.889 (0.004)	35220.5 (19.9)
**O−P**	6.6 (0.5)	192.3 (32.5)	1.4 (1.5)	9.1 (5.8)	0.899 (0.012)	35231.1 (18.4)
**F+P**	8.2 (0.8)	392.1 (33.8)	19.5 (5.0)	17.5 (6.1)	0.826 (0.007)	35259.9 (28.2)
**F−P**	7.6 (0.5)	338 (46)	13.8 (5.4)	20.9 (10.0)	0.832 (0.005)	3526 (37)
**S+P**	3 (0)	0 (0)	0 (0)	10581.1 (73.5)	0.203 (0.003)	4255.9 (14.3)
**S−P**	2.4 (0.5)	0 (0)	0 (0)	13568.8 (146.3)	0.195 (0.002)	3313.8 (43.4)
**R+P**	9.8 (0.9)	189.3 (31.8)	15.8 (4.6)	29.4 (8.0)	0.836 (0.013)	35200.9 (32.1)
**R−P**	6.2 (0.4)	120.2 (16.8)	0.4 (1)	31.1 (7.1)	0.856 (0.019)	35203.2 (30.8)
**E+P**	3 (0)	0 (0)	0 (0)	17174.2 (89.6)	0.205 (0.002)	3997.2 (13.4)
**E−P**	2.3 (0.5)	0 (0)	0 (0)	18926.3 (84.1)	0.195 (0.001)	3261.7 (14.5)
**Cronbach’s *α***	0.994	0.997	0.989	0.999	0.999	0.999

**Table 2.** Results of the ANOVAs based on simulation data for each dependent variable (DV). Across all DVs, there are reliable effects for the presence of police cordons and other variations across scenarios. MSE represents mean squared error.

DV	effect	*F*	MSE	*p*
**max**	police cordons	163.636	0.611	<0.001
	scenario	309.625	0.611	<0.001
	interaction	29.495	0.611	<0.001
**D_4_**	police cordons	116.584	714.914	<0.001
	scenario	744.874	714.914	<0.001
	interaction	32.211	714.914	<0.001
**D_6_**	police cordons	189.153	12.384	<0.001
	scenario	101.953	12.384	<0.001
	interaction	55.153	12.384	<0.001
**TP**	police cordons	5313.469	4230.030	<0.001
	scenario	342056.024	4230.030	<0.001
	interaction	2216.595	4230.030	<0.001
**density**	police cordons	4.183	0.00008	0.044
	scenario	32322.325	0.00008	<0.001
	interaction	9.603	0.00008	<0.001
**congestion**	police cordons	3759.747	735.106	<0.001
	scenario	8110471.825	735.106	<0.001
	interaction	1489.341	735.106	<0.001

Our simulations reproduced the crowding effect in the original scenario at approximately the same time and location it was observed in the video footage. Our complementary results suggest that other crowd management strategies may have led to fewer or no casualties by decreasing density and congestion and increasing throughput. These results support the strategies suggested by Helbing & Mukerji [7]. Specifically, we found that the removal of physical obstacles (i.e. fences and police cordons) and the separation of inflow and outflow substantially reduced the expected number of simulated casualties.

## 3. Virtual reality experiment

**Figure 6.**
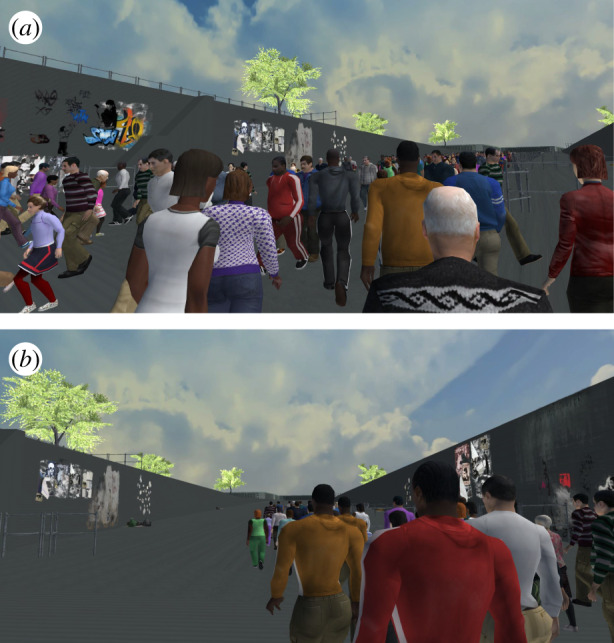
Screenshots from (*a*) the O (original) scenario and (*b*) E–P scenario (additional exit without police cordons) in the virtual reality environment.

While the simulation results provide evidence for the efficacy of these strategies for collective behaviour, they do not by themselves reveal the mechanisms underlying individual reactions to the crowd. In order to compare the best (E–P) and worst (O) scenarios in terms of individuals’ physiological and behavioural responses, we devised a VR experiment in which participants experienced the simulation from a first-person perspective (figure 6). We expected participants in the O condition to be more physiologically aroused than participants in the E–P condition with respect to EDA and HRV. We also expected participants in the O condition to report higher levels of distress and produce more head movements than participants in the E–P condition.

Participants were recruited via the University Registration Center for Participants (www.uast.uzh.ch). A total of 58 participants (27 females; mean age = 27, range = 19–39) participated in the study. Two participants were excluded from the study because of equipment failure. All participants received CHF15 for approximately 45 min of participation. Before each experimental session, participants were given an information sheet and asked to complete an informed consent form. After this consent procedure, the experimenter helped the participant attach the three electrocardiogram electrodes. Next, participants completed the demographics questionnaire, video game experience questionnaire and the pre-test questions from the short stress state questionnaire (SSSQ). The experimenter then attached two electrodes to the participants’ fingers to collect EDA data and placed the head-mounted display (HMD) on the participant’s head. After adjusting the HMD, participants completed a training procedure in which they were asked to look left and right and then look towards a button that was shown at a specific location on the display. After training, a 7 minute nature video was presented to participants in order to record a baseline measure of their physiological activity. During the testing phase, the participants viewed four identical replays of one simulated scenario from a first-person perspective. Each replay was 2 minutes long, and participants had small breaks between replays. The video sequences did not contain any distressful content.

Participants were randomly assigned to one of two groups (O or E–P), each of which represented a scenario from the simulations above. Specifically, we compared replays of the O scenario with replays of the E–P scenario. These simulated scenarios were thus the only between-subject independent variable. Our dependent variables included responses to the video game experience questionnaire, the three subscales of the SSSQ, EDA, HRV, and the magnitude of head movements derived from the gyroscope in the HMD. EDA, HRV and head movement data were aggregated across trials, and all of these measures were compared between the O and E–P scenarios using two-tailed, independent-samples *t*-tests. For more details regarding the experiment methods, please see the electronic supplementary material.

**Figure 7.**
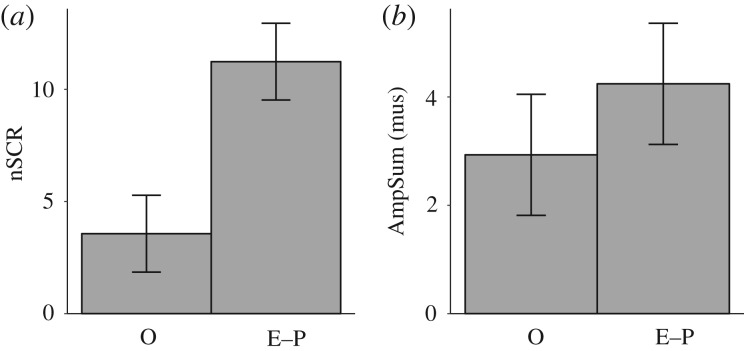
Difference between O and E−P scenarios in terms of (*a*) nSCR and (*b*) AmpSum. The error bars represent the standard error of the difference between the two groups. Although both trends are in the same direction, we only found a significant difference in terms of nSCR (*p* = 0.029).

Results of the VR experiment showed that, for the SSSQ, there were no significant differences between the O and E–P scenarios in terms of distress (O = 0.66 ± 0.71, E−P = 0.54 ± 1.31), *t*(56) = 0.444, s.e. = 0.277, *p* = 0.659; engagement (O = −0.30 ± 0.82, E−P = −0.08 ± 0.97), *t*(56) = −0.93, s.e. = 0.236, *p* = 0.357; or worry (O = −0.26 ± 0.76, E−P = −0.15 ± 0.96), *t*(56) = −0.489, s.e. = 0.227, *p* = 0.627. For EDA, we found a significant difference between the O and E−P scenarios in terms of non-specific skin conductance responses (nSCRs) (O = 3.57 ± 13.91, E−P = 11.24 ± 12.09), *t*(56) = −2.241, s.e. = 3.422, *p* = 0.029, but not in terms of the sum of the amplitude of these peaks (AmpSum) (O = 2.93 ± 10.57, E−P = 4.24 ± 5.76), *t*(56) = −0.586, s.e. = 2.237, *p* = 0.560 (figure 7). This suggests that the frequency (but not the magnitude) of skin conductance responses was higher in the E−P scenario than in the O scenario. For the HRV data, there was no significant difference between the O and E−P scenarios in terms of Log(HF) (O = −0.27 ± 0.79, E−P = −0.12 ± 0.50), *t*(53) = −0.894, s.e. = 0.176, *p* = 0.375. In addition, there was not a significant difference between the O and E−P scenarios in terms of head movements (O = 1150.22 ± 635.17, E−P = 1267.46 ± 527.22), *t*(56) = − 0.765, s.e. = 5.837, *p* = 0.448. During the course of the whole experiment, no participants reported motion sickness or interrupted the procedure because of discomfort.

The results reveal that, when viewing from a first-person perspective, participants had higher nSCRs in the effective intervention group (E−P) than in viewing the original (O) simulation. The simulation of the original crowd disaster may have been less stressful than in reality because, owing to ethical and technical reasons, we could not simulate and present the participants with animations of crowd members falling and stepping on each other. In addition, our design cannot be used to disentangle the effects of crowd movement and visual exposure to the virtual environment. This limitation makes it difficult to interpret the observed effect of management strategies on individual physiological arousal. In order to explain this effect, future research could systematically vary crowd movement and exposure to the environment. However, this approach would require the experimenter to control the viewing direction of the participants during the replays. Future research can also address the possibility of an interaction between motion sickness [48] and stress resulting from differences between the crowd scenarios, although we did not observe any motion sickness in the present study.

## 4. Discussion

In this paper, we present the results of computer simulations and a VR experiment that investigated the effects of possible interventions during the 2010 Love Parade disaster on simulated casualties and physiological arousal in VR. We simulated the original event along with several other scenarios, including the removal of the main fences and/or police cordons, the opening of a side ramp for entering or exiting, the separation of inflow and outflow using the main and side ramps, and the opening of an additional exit near the festival area. These simulations revealed that, compared with the original scenario, all of these interventions led to less congestion, more throughput, and fewer or no simulated casualties. Our simulations provide a mechanism to assess previous disasters and may support event managers in devising strategies to avoid future crowd disasters. Specifically, we demonstrate that crowd simulations based on the SFM and rendered in Unity can be used to determine possible causes of disasters. The application of the SFM was effective for recreating the physical properties and dynamics of the observed crowd. Our introduction of the danger zone metrics allowed us to easily assess the risk level of the simulated event. Our simulation of various management strategies demonstrated that alternative organizational decisions regarding crowd control during the event could have helped to prevent the disaster. In addition, rendering these simulations in Unity may help event managers visualize the effects of specific interventions on the crowd. Our VR experiment revealed that the best (E–P) and worst (O) scenarios significantly differed in terms of the frequency of skin conductance responses.

With respect to the simulations, we found that the most effective strategy for reducing simulated casualties was a combination of removing police cordons and opening an additional exit from the festival area. Most previous research specifically focused on the detection of crowd movement patterns during the Love Parade disaster [8,15,16]. We extended these approaches by simulating several interventions suggested by previous assessments of the organization and operation of this event [7,14]. Our results support the potential of these interventions for saving lives. However, the scope of our finding is limited in terms of using density as a proxy to estimate critical crowded conditions. The simulation of more realistic crowd behaviours such as falling and stepping upon others might help one to establish a greater degree of accuracy in this respect in the future, but there are ethical issues to be considered.

Consistent with Pretorius *et al.* [4], we found that the separation of inflow and outflow (resulting in one primary direction of motion) increases throughput and reduces congestion in the simulated crowd. However, unlike in our study, Pretorius *et al.* [4] did not find any benefit in removing the police cordons. Owing to several differences between the implementations and analyses of the two studies, we cannot identify the exact reason for this difference in results. In our study, the effect of the removal of police cordons was extremely consistent across measures and most scenarios. Indeed, the removal of police cordons in the present simulations decreased the maximum occupation, decreased the number of danger zones, increased throughput, decreased density and decreased congestion. Such an effect is also consistent with recent official expertise [49,50]. Importantly, our study extended that by Pretorius *et al.* [4] by including a measure of simulated casualties based on danger zones. As decreased throughput and increased congestion do not necessarily always result in more danger zones, event planners may adopt metrics such as danger zones in order to gain a better understanding of the potential benefits of particular interventions in the future.

With respect to the VR experiment, we found that viewing a simulation of an effective intervention from a first-person perspective led to higher nSCRs than viewing the original simulation. One possible explanation of our results is that participants in the E–P group moved further along the ramp and were exposed to more variation in the virtual environment than participants in the O group. This additional visual exposure may have increased arousal by inducing engagement or curiosity. The skin conductance results by themselves cannot disentangle these possibilities. Previous research in VR has found that the idle behaviour of a single avatar [34], the presence of a group of avatars [35], and smaller distances between avatars and the observer [36] increased physiological arousal. Furthermore, Llobera *et al.* [36] also found that more moving avatars led to higher physiological arousal, but the number of avatars in their study (maximum four agents) was much lower than in the present study (up to 2000 agents in the original group). Our two VR scenarios were similar with regards to avatar presence and distances, and the main difference between scenarios is the level of congestion. However, congestion was negatively related to avatar motion. Thus, our finding that idle avatars lead to lower physiological arousal may be inconsistent with Fox *et al.* [34]. The VR experiment was also limited in terms of the presence of the simulated crowd in that it did not reproduce crowd turbulence from the original disaster. Hence, the absence of crowd turbulence is likely to have caused participants to experience less stress from the first-person perspective than they would have experienced during the disaster. Future research can focus on explaining the exact mechanisms underlying the significant difference between scenarios by systematically varying crowd parameters (e.g. density, congestion) and environment parameters (e.g. spaciousness, visual noise).

Since the study was limited to a simplified crowd behaviour model, it was not possible to completely reproduce the complex phenomenon and spontaneous crowd movement patterns of the Love Parade crowd disaster. One limitation of our simulations is that rendering simulations in Unity is time intensive and prohibits a large number of replications. As a result, parameters in the SFM were difficult to optimize with respect to the original video data. Nonetheless, conducting 10 repetitions of each scenario allowed us to assess the consistency of each measure and the statistical significance of differences across scenarios. In future studies, one way to overcome this limitation would be to conduct the crowd simulation on a lighter platform and then only render the simulated crowd with optimized trajectories in Unity. Another possible limitation is the manual extraction of crowd data from video footage. In the future, computer vision technology may be used to extract more precise estimates of in- and outflows. Lastly, the simulated crowd is notably less intelligent than members of the real crowd and so does not necessarily reflect the spontaneous behaviours of people reacting to a dangerous situation. Many aspects of the crowd behaviour (e.g. realistic turning behaviour at the corners, crowd turbulence) could have augmented this simplified model and improved the veracity of the simulations. Future research can propose and attempt to validate these more sophisticated models of pedestrian dynamics for large crowds and environments [21,22,51].

Despite these limitations, we demonstrate a novel methodology for the research of crowd disasters and their prevention. To our knowledge, this is the first study to combine simulations and experimentation in VR of a crowd disaster of this complexity and size. We expect that follow-up work will further increase the sophistication and precision of this approach and thereby underline its huge potential and value. In conclusion, the coordination of crowd simulations and VR technologies can help event managers to assess potential dangers more realistically and to make more effective decisions about crowd management strategy in advance.

## Supplementary Material

Supplementary Material

## Supplementary Material

Simulations Data

## Supplementary Material

VR Experiment Data

## Supplementary Material

Video - Density Map

## Supplementary Material

Video - VR

## Data accessibility

The experimental data are available in the electronic supplementary material.
