# Supplementary material for: Assessing crowd management strategies for the 2010 Love Parade disaster using computer simulations and virtual reality
Source: J R Soc Interface. 2020 Jun 10;17(167):20200116. doi: 10.1098/rsif.2020.0116 (PMC7328386; doi:10.1098/rsif.2020.0116)
Supplement: Supplementary Material [file rsif20200116supp1.pdf]

rsif.royalsocietypublishing.org

Research

Article submitted to Journal of the Royal Society Interface

Subject Areas:
computational biology

Keywords:
crowd simulation, crowd disasters, virtual reality, head-mounted display, spatial cognition, physiological arousal

Author for correspondence:
Hantao Zhao
e-mail: hantao.zhao@gess.ethz.ch

Supplementary Material from "Assessing crowd management strategies for the 2010 Love Parade Disaster using computer simulations and virtual reality"

Hantao Zhao[1], Tyler Thrash[1,2,3], Mubbasir Kapadia[4], Katja Wolff[5], Christoph Hölscher[1], Dirk Helbing[6], and Victor R. Schinazi[1,7]

[1]Chair of Cognitive Science, ETH Zürich
[2]Geographic Information Visualization and Analysis, University of Zürich
[3]Digital Society Initiative, University of Zürich
[4]Department of Computer Science, Rutgers University
[5]Interactive Geometry Lab, ETH Zürich
[6]Computational Social Science, ETH Zürich
[7]Department of Psychology, Bond University

THE ROYAL SOCIETY
PUBLISHING

© The Authors. Published by the Royal Society under the terms of the Creative Commons Attribution License http://creativecommons.org/licenses/by/4.0/, which permits unrestricted use, provided the original author and source are credited.

1. Targeting mechanism

Each agent within the crowd was assigned a series of navigation targets (see Figure 1). These potential targets lied along the edges of Regions 1 and 2. The crowd entering the festival area was first directed towards Region 1 and then Region 2. The crowd exiting the festival area was first directed towards Region 2 and then the tunnel to their left or right. To avoid an unrealistic amount of congestion at the corners of each intersection, we defined these navigation targets probabilistically and separately for each agent. The point at which each agent entered or exited an intersection was selected from four discrete targets with equal probability. There were five meters between each of these four potential targets. Figure 1 illustrates the case in which two agents first moved towards the Western edge of Region 1. Agent 1 has an equal probability to choose from *a1* through *a4* on the edge of Area 1 as first navigation point, whereas Agent 2 has an equal probability to choose from *a2* through *a5*. Similar mechanisms were applied for the northern edge of Region 1 and the southern edge of Region 2. The original police cordons were simulated using obstacles that could not be walked through. We tested two other targeting mechanisms, one in which agents attempted to take the shortest path and one in which agents moved directly towards the closest target, but neither of these extremes produced realistic crowd behaviour.

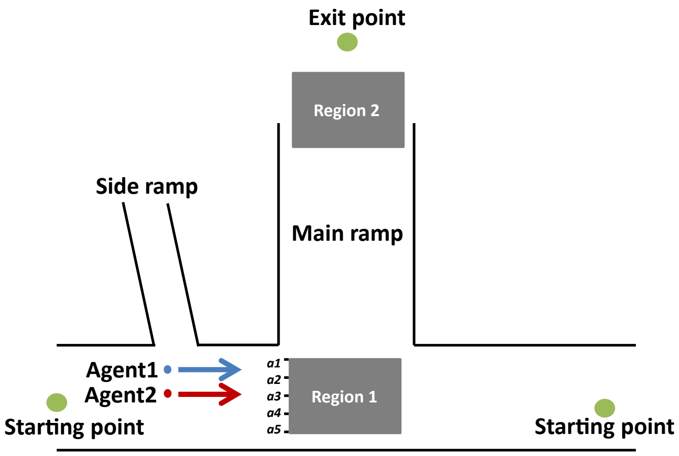

Figure 1. Overview of the targets and trigger areas on the ramp to navigate agents.

2. Methods for VR experiment

(a) Materials

The VR system consisted of a high-end gaming computer (Dell Alienware Area 51 Base; i7-5820K processor at 3.8 GHz overclocked; dual NVIDIA GeForce GTX 1080 video cards; 32 GB of SDRAM; Windows 10 operating system), a head-mounted display (Oculus Rift SDK2; https://www.oculus.com/rift), and physiological data equipment (Powerlab 8/35 recording device with FE116 GSRAmp and FE132 Bio Amp signal amplifiers; https://www.adinstruments.com). Finger electrodes (MLT118F) connected the GSRAmp to the medial phalanges of the index and ring fingers of each participant's non-dominant hand. In addition, we used three disposable electrocardiogram electrodes (MLA1010) to collect data for HRV analyses. These electrodes were placed on the second intercostal space below the middle of the clavicle on the right and left side of the chest and below the ninth left rib.

The VR experiment was implemented using Unity and the Experiments in Virtual Environments (EVE) framework [1,2]. The virtual environment was based on the environment described above. Additional decals and game textures from the Unity Asset Store (https://assetstore.unity.com/) were placed along the walls of the tunnel and main ramp in order to improve optic flow and provide a more realistic setting. In addition, we used LabChart 8.14 (https://www.adinstruments.com/) for collecting the physiological data, Matlab R2017a (https:// www.mathworks.com) for exporting and segmenting the physiological data, Ledalab 3.49 (http://www. ledalab.de) for processing the EDA data, and Kubios HRV 3.1 (https://www.kubios.com) for analysing the HRV data.

Participants have read and signed consent in all experiments. Participants were recruited via the University Registration Center for Study Participants (http://uast.uzh.ch). Participants completed a demographics questionnaire, a video game experience questionnaire, and the SSSQ. The demographics questionnaire contained questions regarding participants' gender, handedness, vision, and experience with head-mounted displays. The video game experience questionnaire asked participants to judge the number of hours per week that they had played video games in each of several categories (e.g., first-person shooters, sports games) [3]. The Short Stress State Questionnaire (SSSQ) consists of 24 pre- and post-test items that can be divided into three subscales (i.e., distress, engagement, and worry) [4]. The distress subscale of the SSSQ is an indicator of positive arousal and negative valence, while the engagement subscale refers to positive arousal and positive valence. The worry subscale represents self-focused attention and intrusive thoughts.

(b) Design and analyses

Our physiological data included both electrodermal activity (EDA) and heart rate variability (HRV). While EDA reflects sympathetic arousal, (normal) HRV results from a balance between sympathetic and parasympathetic activity [5]. For each trial, we exported the EDA data from LabChart to Ledalab. In Ledalab, this data was then downsampled from 1000 Hz to 10 Hz and visually inspected for artefacts. No artefacts were detected. We used Continuous Decomposition Analysis in order to extract the number of peaks in the EDA data (nSCR) and the sum of the amplitude of these peaks (AmpSum) [6]. Experiments in which the timing of the evocative stimulus depends on participant behaviour often measure the frequency of nSCR [7]. For this analysis, the parameter estimation algorithm was optimized twice in order to model the impulse response function. The threshold for each individual peak was $0.01\mu s$. After subtracting the baseline, both nSCR and AmpSum should represent overall reactivity for each trial.

HRV can be assessed in time or frequency domains. Researchers often consider decreases in the high frequency band (i.e., 0.15 to 0.40 Hz) as an indicator of stress [8]. The electrocardiogram data was sampled at 1000 Hz and exported from LabChart to Kubios HRV for analysis. After automatic beat detection and artefact correction, the time series of interbeat intervals for each trial were visually inspected for additional artefacts. In total, we removed one participant completely and 4 trials (.02%) from the remaining 57 participants due to missing or misaligned beats [9]. Each participant had at least three trials for further analysis. This data was then processed using a very low threshold filter (0.45 sec) and a smoothness prior filter for detrending (λ = 500, cut-off frequency = 0.035 Hz). We then used a Fast Fourier transform that was focused on power in the high frequency range (0.15 to 0.4 Hz) that typically reflects parasympathetic nervous system activity [10,11]. The absolute values for high frequency power were natural log transformed (Log(HF)) in order to produce a measure of stress, panic, anxiety, and/or worry.

References

1. Grübel J, Thrash T, Hölscher C, Schinazi VR. 2017 Evaluation of a conceptual framework for predicting navigation performance in virtual reality. *PloS one* **12**, e0184682.

2. Weibel RP, Grübel J, Zhao H, Thrash T, Meloni D, Hölscher C, Schinazi VR. 2018 Virtual Reality Experiments with Physiological Measures. *JoVE (Journal of Visualized Experiments)* p. e58318.
3. Green CS, Kattner F, Eichenbaum A, Bediou B, Adams DM, Mayer RE, Bavelier D. 2017 Playing some video games but not others is related to cognitive abilities: A critique of Unsworth et al.(2015). *Psychological science* **28**, 679–682.
4. Helton WS. 2004 Validation of a short stress state questionnaire. In *Proceedings of the Human Factors and Ergonomics Society Annual Meeting* vol. 48 pp. 1238–1242. SAGE Publications.
5. Acharya UR, Joseph KP, Kannathal N, Lim CM, Suri JS. 2006 Heart rate variability: a review. *Medical and biological engineering and computing* **44**, 1031–1051.
6. Benedek M, Kaernbach C. 2010 A continuous measure of phasic electrodermal activity. *Journal of neuroscience methods* **190**, 80–91.
7. Boucsein W, Fowles DC, Grimnes S, Ben-Shakhar G, Roth WT, Dawson ME, Filion DL. 2012 Publication recommendations for electrodermal measurements. *Psychophysiology* **49**, 1017–1034.
8. Kim HG, Cheon EJ, Bai DS, Lee YH, Koo BH. 2018 Stress and heart rate variability: A meta-analysis and review of the literature. *Psychiatry investigation* **15**, 235.
9. Camm AJ, Malik M, Bigger JT, Breithardt G, Cerutti S, Cohen RJ, Coumel P, Fallen EL, Kennedy HL, Kleiger R et al.. 1996 Heart rate variability: standards of measurement, physiological interpretation and clinical use. Task Force of the European Society of Cardiology and the North American Society of Pacing and Electrophysiology. .
10. Berntson GG, Thomas Bigger Jr J, Eckberg DL, Grossman P, Kaufmann PG, Malik M, Nagaraja HN, Porges SW, Saul JP, Stone PH et al.. 1997 Heart rate variability: origins, methods, and interpretive caveats. *Psychophysiology* **34**, 623–648.
11. Rawenwaaij-Arts C, Kallee L, Hopman J et al.. 1993 Task Force of the European Society of Cardiology and the North American Society of Pacing and Electrophysiology. Heart rate variability. Standards of measurement, physiologic interpretation, and clinical use. Circulation 1996; 93: 1043-1065.. *Intern. Med* **118**, 436–447.
